# Comparison of autonomous sensory meridian response and binaural auditory beats effects on stress reduction: a pilot study

**DOI:** 10.1038/s41598-022-24120-w

**Published:** 2022-11-14

**Authors:** Minji Lee, Hyuk Joo Lee, Junseok Ahn, Jung Kyung Hong, In-Young Yoon

**Affiliations:** 1 Department of Neuropsychiatry, Seoul National University Bundang Hospital, Seongnam, 13620 Korea; 2 Department of Public Medical Service, Seoul National University Bundang Hospital, Seongnam, 13620 Korea; 3 Department of Psychiatry, Ulsan University Hospital, Ulsan, 44033 Korea; 4 Department of Psychiatry, Seoul National University College of Medicine, Seoul, 03080 Korea

**Keywords:** Stress and resilience, Psychiatric disorders

## Abstract

This study aimed to compare the effects of Autonomous sensory meridian response (ASMR) and binaural beat (BB) on stress reduction, and to determine whether ASMR and BB can induce changes in quantitative electroencephalography (QEEG). A double-blind randomized trial was conducted. Subjects with stress were recruited considering their perceived stress scale (PSS), Beck depression inventory-II (BDI-II), insomnia severity index (ISI), and state-trait anxiety inventory-state anxiety (STAI-S) scores. Subjects listened to ASMR or BB with music (8 Hz for daytime, 5 Hz for nighttime) for 15 min in daytime and 30 min before going to sleep for 3 weeks. QEEG was measured before and after the intervention. Seventy-six participants (57 female, mean age = 46.12 ± 12.01) finished the trial. After the intervention, PSS, BDI-II, ISI, STAI-S, and PSQI scores improved significantly in both groups. BDI-II and ISI mean scores were normalized in both groups after the intervention. Changes of absolute beta and high beta power in the ASMR group were larger than those in the BB group (*p* = 0.026*, p* = 0.040, respectively). Both ASMR and BB are equally effective in reducing stress levels. Unlike BB, ASMR can lead to an increase in beta and high beta waves associated with cortical arousal.

## Introduction

Stress is a transactional process that emerges from real or perceived environmental demands, depending on the availability of an individual’s adaptive coping strategy^1^. Moderate level of stress is essential for survival, but excessively long and repeated exposure to stressors can lead to an exhaustion stage beyond adequate tension^2^. In the exhaustion stage, stress can cause vague somatic symptoms and immune deficiency that promote the development of allergic reactions, asthma, and cancer^3–5^. In addition, stress is a clear trigger for psychiatric disorders such as mood and anxiety disorders^6,7^.

Various attempts have been made to reduce the effects of stress using sounds. Autonomous sensory meridian response (ASMR) is a sensory-emotional phenomenon in which specific auditory, audiovisual, or tactile stimuli elicit tingling sensations on the scalp, neck, and arms^8–10^. Sensorimotor experiences associated with ASMR are often accompanied by a sense of being calm, which can be helpful in reducing stress. Paszkiel et al. found that listening to ASMR calms subjects much faster than silence by measuring stress levels with EEG, blood pressure, pulse, and questionnaire^9^. Barratt & Davis’s study on 475 participants, 80% reported that ASMR improved their mood^8^. However, responses to ASMR may different from person to person. Only people who are sensitive to ASMR experience a positive effect such as calmness and excitement^10^. In another study, depressive feelings decreased in those experiencing tingles after watching the ASMR video compared with individuals without tingles. In addition, all participants experienced a decrease in HR when watching the ASMR video, regardless of whether they felt tingles^11^. According to these results, mood symptoms improved only when individual is sensitive to ASMR.

There is also an increased interest in the therapeutic effect of binaural beat (BB) auditory stimulation. Binaural beats are perceptual phenomena that occur when two similar, pure tones are delivered to each ear separately^12^. These beats are thought to originate from the medial nucleus of the superior olive complex which receives bilateral input for the first time^13^. BB also seems to modulate mood, pain, perception, and insomnia. In a meta-analysis study by Garcia-Argibay et al., BB showed efficacy on memory, attention, anxiety, and analgesia to a modest degree^14^. In addition, Padmanabhan et al. reported that listening to BB before surgery was more effective in reducing anxiety than listening to music^15^.

Despite the reported effects of ASMR and BB on stress reduction and their widespread use, there were no studies directly comparing the effectiveness of the two. This study aimed to compare the stress-reducing effect of ASMR and BB, and to determine which is more acceptable. In comparing stress reduction between ASMR and BB, we used both subjective and objective measurements. We evaluated stress perception, depression, anxiety and insomnia, and we also performed quantitative electroencephalogram (QEEG) analysis.

## Results

**Figure 1:**
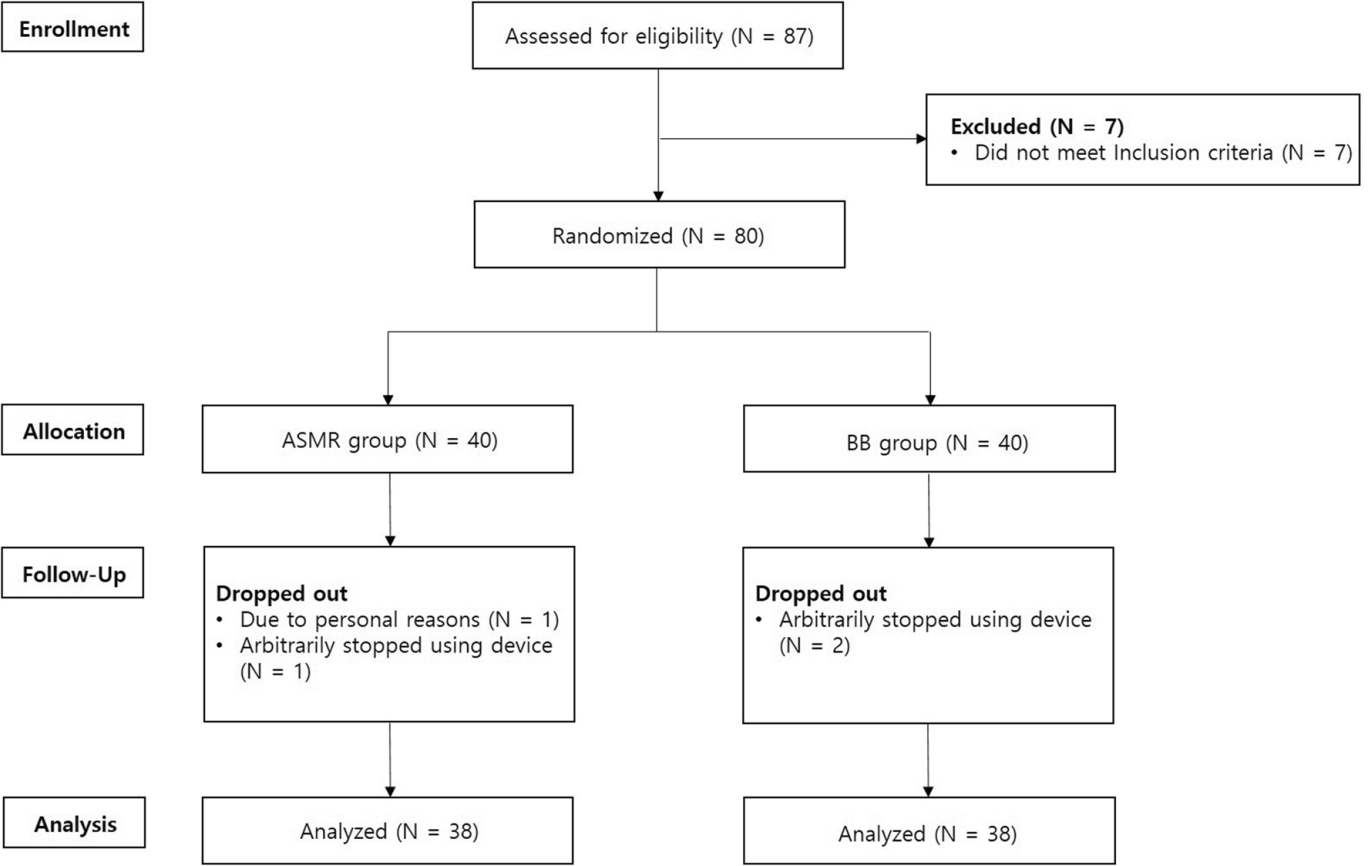
Flow chart summary of the participants. *ASMR*: Autonomous sensory meridian response, *BB*: Binaural beat.

A total of 87 people were screened, and 80 people met the screening criteria and participated in the study. Forty participants were assigned to the BB group, and 40 participants were assigned to the ASMR group by stratified permuted block randomization. One subject dropped out due to personal reasons and 3 subjects arbitrarily stopped while using the device. Seventy-six subjects completed the test (Fig. 1).

There was no significant difference in demographic factors such as sex, age, and BMI, and in sleep parameters measured through the sleep diary. The overall compliance of days and nights use of the device was over 90%, and there was no difference in compliance between the two groups (Table 1). In the analysis of perceived stress scale (PSS), Beck depression inventory-II (BDI-II), insomnia severity index (ISI), state-trait anxiety inventory-state anxiety (STAI-S), Pittsburgh Sleep Quality Index (PSQI), and Quality of Life scale abbreviated version (QOL-BREF) scores, significant improvement was reported after using the device in both groups. Estimated effect sizes were large enough in both groups, in PSS, BDI-II, ISI, STAI-S, PSQI, and QOL-BREF scores (Cohen *d* > 0.8). In BDI-II and ISI, mean scores were improved to normal range in both groups. No significant group-by-time interaction was found in PSS, BDI-II, ISI, STAI-S, PSQI, and QOL-BREF scores at 3 weeks between the two groups. Sleep latency, total sleep time, time in bed were significantly improved in both group with sleep efficiency being increased only in the BB group (p < 0.02). There was no significant group-by-time interaction in sleep parameters (Table 2).

**Table 1:** Demographic characteristics of participants (N = 76).

	Total	ASMR group	BB group	*p-value*
n	76	38	38	
Male (%)	19 (25)	10 (26)	9 (24)	0.794
Age (years)	46.12 ± 12.01	46.00 ± 11.42	46.24 ± 12.71	0.932
BMI	23.41 ± 3.83	24.11 ± 4.50	22.72 ± 2.93	0.115
PSS	22.76 ± 3.13	22.63 ± 3.54	22.89 ± 2.69	0.717
BDI-II	23.03 ± 8.52	24.45 ± 8.21	21.61 ± 8.69	0.147
ISI	12.66 ± 4.47	12.66 ± 4.86	12.66 ± 4.12	1.000
STAI-S	53.99 (8.85)	54.55 ± 10.14	53.42 ± 7.42	0.581
PSQI	8.78 ± 2.59	8.84 ± 2.80	8.71 ± 2.40	0.826
QOL-BREF	76.46 ± 12.88	74.55 ± 13.21	78.37 ± 12.42	0.198
SL (min)	42.50 ± 27.34	40.66 ± 22.18	44.34 ± 22.18	0.953
TST (hr)	5.73 ± 1.05	5.58 ± 1.13	5.89 ± 0.94	0.259
TIB (hr)	6.57 ± 1.21	6.33 ± 1.12	6.81 ± 1.27	0.166
SE (%)	87.35 ± 9.91	87.53 ± 10.90	87.17 ± 8.95	0.553
Day use compliance	92.11 ± 12.11	93.11 ± 10.89	91.98 ± 14.86	0.474
Night use compliance	92.54 ± 12.95	93.11 ± 10.89	91.98 ± 14.86	0.707
Overall compliance	92.32 ± 11.89	93.11 ± 10.47	91.54 ± 13.26	0.569

Data represent *N (%)* for sex and *mean* ± *standard deviation* for others. The independent t-test or Mann–Whitney U test was performed for numerical variables and the chi-squared test was performed for categorical variables. *PSS*: Perceived stress scale, *BDI-II*: Beck depression inventory II, *ISI*: Insomnia severity index, *STAI-S*: State-trait anxiety inventory-state anxiety, *PSQI*: Pittsburgh Sleep Quality Index, *QOL-BREF*: Quality of Life scale abbreviated version, *SL*: Sleep latency, *TST*: Total sleep time, *TIB*: Time in bed, *SE*: Sleep efficiency, *ASMR*: Autonomous sensory meridian response, *BB*: Binaural beat.

**Table 2:** Mean comparison of questionnaires and sleep parameters before and after application use.

	*ASMR group (n* = *38)*				*BB group (n* = *38)*				Group X Time *P*	Time *p*	Group *p*
	Pre	Post	*p*	Cohen’s d	Pre	Post	*p*	Cohen’s d			
	Mean ± SD	Mean ± SD			Mean ± SD	Mean ± SD					
PSS	22.63 ± 3.54	16.16 ± 3.28	** < 0.001**	1.89	22.89 ± 2.70	16.74 ± 3.46	** < 0.001**	1.98	0.72	** < 0.01**	0.49
BDI-II	24.45 ± 8.21	12.32 ± 6.48	** < 0.001**	1.63	21.61 ± 8.69	11.29 ± 7.79	** < 0.001**	1.25	0.38	** < 0.01**	0.19
ISI	12.66 ± 4.86	6.76 ± 4.08	** < 0.001**	1.31	12.66 ± 4.12	6.32 ± 4.23	** < 0.001**	1.52	0.76	** < 0.01**	0.78
STAI-S	54.55 ± 10.14	42.82 ± 9.22	** < 0.001**	1.21	53.42 ± 7.42	42.68 ± 8.93	** < 0.001**	1.31	0.65	** < 0.01**	0.72
PSQI	8.84 ± 2.80	5.47 ± 2.63	** < 0.001**	1.24	8.71 ± 2.40	5.24 ± 2.19	** < 0.001**	1.51	0.87	** < 0.01**	0.70
QOL-BREF	74.55 ± 13.21	92.29 ± 14.08	** < 0.001**	1.30	78.37 ± 12.42	91.45 ± 15.85	** < 0.001**	0.92	0.15	** < 0.01**	0.59
SL (min)	40.66 ± 22.18	25.13 ± 17.61	** < 0.001**	0.78	44.34 ± 22.18	27.21 ± 28.56	** < 0.001**	0.67	0.73	** < 0.01**	0.60
TST (hr)	5.58 ± 1.13	6.38 ± 1.14	** < 0.001**	0.70	5.89 ± 0.94	6.76 ± 1.27	** < 0.001**	0.78	0.83	** < 0.01**	0.11
TIB (hr)	6.33 ± 1.12	7.01 ± 1.07	** < 0.001**	0.62	6.81 ± 1.27	7.41 ± 1.22	**0.02**	0.48	0.76	** < 0.01**	0.06
SE (%)	87.53 ± 10.90	90.45 ± 9.86	0.12	0.28	87.17 ± 8.95	90.87 ± 8.58	**0.02**	0.42	0.74	** < 0.01**	0.99

Significant values are in bold.

Paired t-test was performed to examine within-group differences. Cohen’s d was calculated to estimate the effect size. Repeated measures analysis of variance (RM ANOVA) was used to examine group-by-time interaction. *PSS*: Perceived stress scale, *BDI-II*: Beck depression inventory II, *ISI*: Insomnia severity index, *STAI-S*: State-trait anxiety inventory-state anxiety, *PSQI*: Pittsburgh Sleep Quality Index, *QOL-BREF*: Quality of Life scale abbreviated version, *SL*: Sleep latency, *TST*: Total sleep time, *TIB*: Time in bed, *SE*: Sleep efficiency, *ASMR*: Autonomous sensory meridian response, *BB*: Binaural beat.

**Figure 2:**
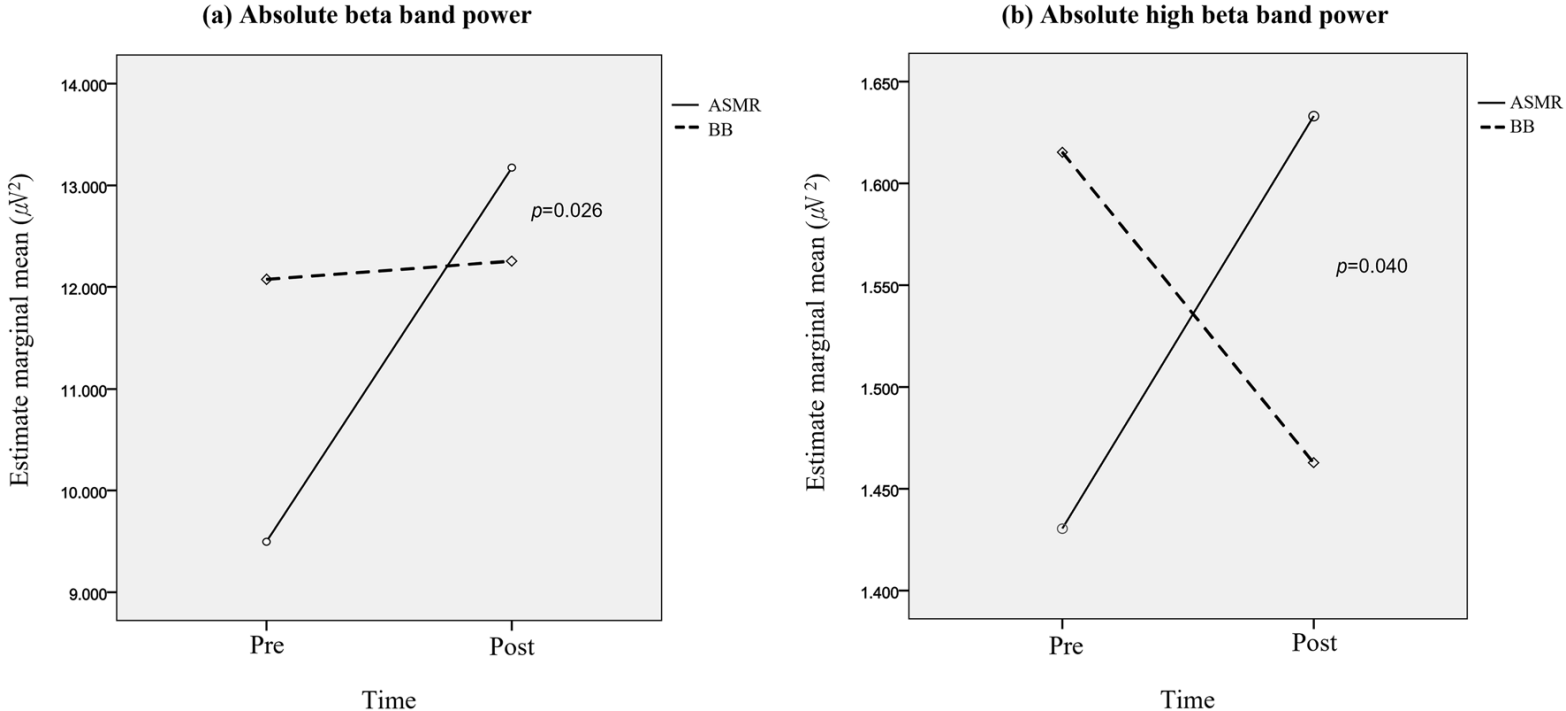
Differences in the changes of absolute power between the ASMR and BB interventions. Group-by-time interaction in absolute beta band power (**a**) and absolute high beta band power (**b**). *ASMR*: Autonomous sensory meridian response, *BB*: Binaural beat.

In the case of absolute band power extracted from the QEEG test, absolute beta and high beta band power in the ASMR group increased (beta: 9.50 ± 4.49 to 13.17 ± 7.51, *p* = 0.002, high beta: 1.43 ± 0.56 to 1.63 ± 0.70, *p* = 0.034). There was a significant group-by-time interaction in absolute beta and high beta band power (beta: *p* = 0.026, high beta: *p* = 0.040) (Fig. 2). In the relative power, there was no significant difference in the change between the two groups before and after using the sound sources (Table 3).

**Table 3:** Band power comparison of before and after application use measured by quantified electroencephalogram.

		ASMR group (n = 38)			BB group (n = 38)			Group X Time *P*	Time *P*	Group *P*
		Pre	Post	*p*	Pre	Post	*p*			
		Mean ± SD	Mean ± SD		Mean ± SD	Mean ± SD				
Absolute	Delta	15.88 ± 7.77	16.64 ± 12.12	0.705	16.68 ± 13.97	17.61 ± 13.44	0.683	0.956	0.576	0.705
	Theta	11.27 ± 7.51	11.67 ± 8.03	0.675	11.45 ± 7.38	12.79 ± 9.93	0.279	0.544	0.263	0.709
	Alpha	17.29 ± 20.23	26.09 ± 25.92	**0.006**	28.98 ± 32.87	30.01 ± 27.44	0.852	0.220	0.122	0.148
	Beta	9.50 ± 4.49	13.17 ± 7.51	**0.002**	12.08 ± 7.87	12.25 ± 5.89	0.868	**0.026**	**0.015**	0.525
	High beta	1.43 ± 0.56	1.63 ± 0.70	**0.034**	1.62 ± 1.15	1.46 ± 0.74	0.292	**0.040**	0.769	0.965
Relative	Delta	29.81 ± 9.80	24.56 ± 10.56	**0.004**	25.71 ± 10.36	23.15 ± 9.30	0.239	0.329	**0.006**	0.139
	Theta	20.29 ± 6.30	17.09 ± 7.07	**0.007**	17.07 ± 6.82	16.69 ± 6.90	0.753	0.087	**0.031**	0.176
	Alpha	20.99 ± 12.25	26.91 ± 14.43	**0.003**	27.68 ± 14.61	31.90 ± 13.52	0.163	0.630	**0.005**	**0.029**
	Beta	18.73 ± 6.82	20.93 ± 7.93	**0.045**	18.24 ± 6.73	18.35 ± 7.40	0.866	0.098	0.067	0.322
	High beta	3.02 ± 1.08	2.93 ± 1.45	0.616	2.75 ± 1.29	2.45 ± 1.35	**0.026**	0.349	0.082	0.184

Significant values are in bold.

Paired t-test was performed to examine within-group differences. Cohen’s d was calculated to estimate the effect size. Repeated measures analysis of variance (RM ANOVA) was used to examine group-by-time interaction. *ASMR*: Autonomous sensory meridian response, *BB*: Binaural beat.

Two participants in the ASMR group and one in the BB group complained of a mild headache but improved after follow-up without any treatment. No other adverse event was reported over the trial period.

## Discussion

This study investigated the effects of ASMR and BB on stress reduction and the difference in QEEG after the intervention. In the analysis of PSS, BDI-II, ISI, STAI-S, PSQI, and QOL-BREF, subjective improvement in stress, anxiety, insomnia, and quality of life were reported in both groups after using the sound sources for 3 weeks. In the case of absolute band power extracted through the QEEG test, beta and high beta power increased in the ASMR group, and there were differences in the change of beta and high beta power between the groups. However, there was no significant difference in the relative power between the ASMR group and BB group.

In both the ASMR and BB groups, subjective stress measured by questionnaires decreased to a similar extent. In the previous studies, the stress reduction effect between music and ASMR was the same, and produces a pleasant state of relaxation^9,16^. Mindfulness may involve spending specific time engaging in ASMR and therefore, when engaging in ASMR, the participant takes time to focus on the positive feelings triggered by the stimuli. This behavior is very similar to mindfulness practices, which many studies have shown to improve both mental and physical well-being^8^. A previous study comparing music and BB with music also showed similar effects in reducing anxiety^15^. In our previous study, BB with music improved sleep to a similar extent to pure music^17^. BB can reduce anxiety even without masking it with comfortable sounds^18^. Similarly, listening to delta and theta wave BB can reduce anxiety^19^. Binaural-beat stimulation can cause central nervous system arousal that affects behavior and mood^20^. Both the ASMR and binaural beats reduced stress levels, particularly in the BDI-II and ISI, the average scores returned to normal.

In the QEEG, the absolute beta and high beta band power in the ASMR group increased. These results are also consistent with previous studies that showed an increase in beta power in the entire group of participants who watched ASMR videos^11^. This can be explained in two ways. First, ASMR's characteristics related to tingling sensations can influence the rise of beta waves. Sensorimotor processing^21^, auditory processing^22^, and observation of the tactile input involve beta modulation^23^. All these processes are specifically linked to the processing of the ASMR stimuli and the perception of ASMR tingling. Second, ASMR may be perceived as an annoying stimulus. ASMR is only enjoyable for those who have experienced it and may even be unpleasant for those who have not^8^. The beta waves are associated with emotions^24^, and can also be increased in stressful situations^25^. In functional magnetic resonance imaging (fMRI) study, while classical music and ASMR display significant activation in similar areas, ASMR shows activation in more areas, primarily in the medial prefrontal cortex^16^. In an electroencephalogram study, ASMR experience was associated with increased alpha activity in the frontal sites^26^. Considering the inhibitory effect of the alpha, the enhanced alpha may reflect a suppression of distractors and an active effort to disengage attention from the stimuli^27^. In our study, the alpha wave was also increased in the ASMR group although there were no significant differences between the groups. Contrary to our results of increased alpha activity, Engelbregt et al. reported that alpha power was decreased in healthy young adults when ASMR-sensitive participants watched ASMR videos^11^. This discrepancy of the ASMR effect on the alpha band might be explained by the individual sensitivity to ASMR and participants’ characteristics, as our study was conducted in adults under stress. In another study, ASMR participants showed increased sensorimotor rhythm activity (12.5–15 Hz) when compared to the control participants, which was shown as an increase in beta waves in our study^28^. Beta waves are well known to reflect cortical hyper arousal on quantified EEGs^29^. In light of this, ASMR has an overall alerting effect.

In our study, the BB group failed to show entrainment effects. Although the exact mechanism is not known yet, BB is known to entrain cortical activity at a specific frequency and cross-frequency modulations. Theoretically, it refers to the tendency of the human brain to change major brain frequencies toward the frequency of external stimuli. This phenomenon has been reported in several studies, including changes in delta^30^, theta^30,31^, and gamma^32^, and not for beta^33^, but there are inconsistencies in the literature. Therefore, even though we could not find the evidence of entrainment effect in this study, it may be premature to conclude that binaural beats are ineffective given that various factors can influence entrainment. In this study, we expected an increase in alpha wave through 8 Hz BB for daytime and theta wave through 5 Hz BB for nighttime. Alpha wave during daytime may be related with alertness and, theta wave during nighttime can be correlated with sedation and good sleep. There is a possibility that the entrainment effect of BB was affected by different frequencies. In previous studies, no entrainment effect was observed in alpha waves^34,35^. However, some studies have observed an entrainment effect in theta wave^30,31^, while others have not observed such an effect^34,35^. Interestingly, most of the experiments that used multiple different frequencies to one subject did not show entrainment effect^34–36^. In our study, it is possible that the entrainment effect was not observed in our study due to interference between the alpha and theta waves.

The present study has several limitations. First, for the convenience of users, we prepared three different sounds for the ASMR group and BB group. Since ASMR is only pleasant for some experienced individuals and unpleasant for non-experienced individuals^8,37,38^, it was necessary to allow participants to listen to their preferred sound among the three sounds provided. Their brain responses may differ because they heard different sounds. However, as many subjects listened to more than one sound during the intervention period without exactly reporting the use time of each sound, analysis according to different sounds was not possible. Second, there may have been heterogeneities in the characteristics of people with stress. In our study, PSS, BDI-II, STAI-S, and ISI scores had positive correlations. People with stress may be a heterogeneous group with symptoms such as depression, anxiety, and insomnia. The heterogeneity of these groups may have made it difficult to determine whether ASMR and BB directly affected stress, depression, anxiety, or sleep. Third, since QEEG was not measured while listening to sounds, the effect of brain entrainment by BB could not be confirmed. It is possible that BB had a brain entrainment effect, but it could not be maintained for a long time. To verify whether entrainment was not achieved or if it was short-lived, a follow-up study is required. Fourth, compliance may not be accurate as it is self-reported, but during the study period, we checked the compliance over the phone every week, and encouraged subjects to keep a sleep diary about the use of the device. Lastly, first recruitment started before the study was registered. The Korean Clinical Research Information Service (CRiS) allows retrospective registration of clinical trials, but we should have registered the study protocol before beginning the clinical trial in order to meet international standards.

## Conclusion

To our knowledge, this study is the first attempt to compare the effects of ASMR and BB on stress reduction with a randomized, double-blinded design. ASMR and BB are equally effective in reducing stress and are safe methods for stress reduction. This study is meaningful in that ASMR and BB improved the PSS, BDI-II, ISI, STAI-S, PSQI, and QOL-BREF scales considerably without major side effects. ASMR increases absolute beta and high beta band power associated with cortical arousal. In this study, no brain entrainment effects were observed on BB, and further studies are necessary.

## Methods

### Participants

From June 2020 to February 2021, subjects aged 19 to 65 years with stress were recruited from the community. We placed an approved advertisement about the clinical trial on local newspapers, subways, and noticeboards of Seoul National University Bundang Hospital. Individuals with stress were defined according to their PSS, BDI-II, ISI, and STAI-S scores. Exclusion criteria included; (1) history of neurological deficit (dementia, brain injury, and seizure disorder), (2) presence of major psychiatric disorders (3) persons with acute stress (after the death of family, restraint, divorce, separation, debt, delinquency, affair, business failure, serious illness, marriage, etc.) within 4 weeks before the trial, (4) receiving cognitive behavioral therapy, biofeedback, neurofeedback, or meditation therapy within 1 year before the trial (5) taking psychotropic drugs, oral steroids or corticosteroids, health functional foods related to stress and sleep improvement within 2 weeks. Using the Beck depression scale as a measure of symptom improvement after the intervention, the minimum number of subjects was 36 for each group for the independent sample t-test to produce effect size 0.7 and power 0.9 under the 5% significance level.

All participants provided written informed consent. The trial was approved by the Institutional Review Board of Seoul National University Bundang Hospital (IRB number, B-2006/616–304) and registered at the Clinical Research Information Service (CRiS registry number, KCT0007633, 09/08/2022). First recruitment date was June 23rd 2020. Guidelines and regulations were followed when performing all methods.

### Measures

We performed the PSS, BDI-II, ISI, STAI-S, PSQI, and QOL-BREF questionnaires before and after the intervention. We also measured baseline and post-intervention QEEG to examine the objective effects of ASMR and BB.

#### Questionnaires

Participants filled out a PSS, BDI-II, ISI, and STAI-S questionnaires. Stress was defined according to PSS, BDI-II, ISI, and STAI-S scores. PSS is one of the most widely used stress scales that evaluates the subject's perceived stress experience on a 5-point Likert scale (0–4 points) during the past month^39^. Since the tool was not developed for diagnostic purposes, the cut-off points are not separately presented, but 0–13 points are classified as low, 14–26 points as moderate, and 27–40 points as severe stress. Those with a PSS score of 14 or higher were determined as stressful. As stress can induce depressed mood, insomnia, and anxiety, we also included any one of the following in the criteria: BDI-II 20-45, ISI 8-21, or STAI-S > = 39. The BDI-II is a self-report questionnaire assessing depressive symptoms in the past 2 weeks^15^. It contains 21 items, and the total score ranges from 0 to 63. Scores of 0–13 were considered as not depressed, 14–19 as mildly depressed, 20–28 as moderately depressed, and 29–63 as severely depressed^40^. The ISI is a 7-item self-report questionnaire assessing subjective insomnia. Its scores range from 0 to 28, and interpretation is as follows: 0–7 as the absence of insomnia, 8–14 as sub-threshold insomnia, 15–21 as moderate insomnia, and 22–28 as severe insomnia^41^. The STAI-S is a self-report screening tool with 20 items to measure state anxiety, on a 4-point Likert scale. A cut-off point of 39 has been suggested for clinically significant symptoms of anxiety^42^.

#### Quantitative electroencephalogram (QEEG)

All subjects underwent QEEG (64-channel NeuroScan SynAmps, Compumedics, North Caroline, USA) with standard electrodes. We recorded EEG for 7 min with eyes closed state followed by a 1-min recording with eyes open state and 7 min with eyes closed state in a sitting position. All the subjects were instructed to keep their positions without movement to prevent muscle artifacts. We encouraged subjects to remain awake during the EEG measurements. EEG electrodes were placed according to the international 10–20 system with an average reference (FP1, FP2, F3, F4, F7, F8, C3, C4, P3, P4, T3, T4, T5, T6, O1, O2, average reference) by using electrodes attached to an elastic cap (Easycap, EASYCAP GmbH, Munich, Germany). The impedance of all the electrodes was below 5 kΩ. EEG signals were sampled at 1000 Hz and digitalized by using 24-bit actiCHamp DC amplifier and BrainVision Recorder (Brain Products Gmbh, Gliching, Germany). The high pass filter was set to 100 Hz with the low pass filter set to 0.3 Hz. An artifact-free 120-s EEG recording with eyes closed (24 epochs of 5-s EEG segments) was selected by visual analysis. Artifacts such as muscle activity, small body movements, eyelids movements, and micro-sleep were not included. Fast Fourier transform was applied to spectral analysis. Absolute power values of five bands at each electrode were computed: delta (1.0–4.0 Hz), theta (4.0–8.0 Hz), alpha (8.0–12.0 Hz), beta (12.0–25.0 Hz), and high beta (25.0–30.0 Hz). Relative power values were computed as the percentage of absolute power. All preprocessing and analysis procedures were conducted using NeuroGuide software 2.0 (Applied Neuroscience, Inc., Florida, USA).

### Intervention

Subjects in the ASMR group listened to the ASMR sound. Since the ASMR sound can be uncomfortable for some people, we prepared three types of sounds: (1) scraping non-woven fabric sound, (2) birdsongs, and (3) white noise. Participants were allowed to choose one preferred sound and listened to it for 15 min during the daytime (before 2 PM) and 30 min at nighttime (before going to bed) for 3 weeks. The music was automatically stopped after the intended periods by a predetermined setting in the application. In the morning, each subject completed a sleep diary, which included information about sleep latency, time in bed, awake after sleep onset, listening time, kind of music, and daytime sleepiness.

In the BB group, we used an application manufactured by Dlogixs, Inc. (Gyeonggi-do, South Korea), which produces a binaural beat, to stimulate brainwave production. In contrast to other devices that produce monotonous sounds with binaural beats, this product allows users to hear music without being aware of distortions of sound sources and resistance to monotonous sound. The audio apparatus shifts the frequency band as much as the target frequency from the original audio sound in real-time to generate binaural beats. We targeted an 8 Hz binaural beat for daytime and a 5 Hz binaural beat for nighttime. Three kinds of music were selected for the trial: (1) Debussy—Arabesque No. 1, (2) Mozart—Concerto for flute, harp & orchestra in C major, K. 299 (K. 297c), and (3) Mozart—Piano Concerto No. 21 in C, K 467, Elvira Madigan. The BB group participants listened to BB for 15 min during the daytime and 30 min at nighttime for 3 weeks. The device was used in an individual's home, and during the daytime, listening was permitted outside the home according to their schedule. We judged good compliance as listening to the sound for at least 70% of the sessions during the 3-week study period, based on the diaries. We contacted the subjects with phone call at week 1 and week 2 to ensure they were using the device properly. Same sets of smartphones and Bluetooth wireless earphones were provided to both groups.

### Statistical analysis

SPSS version 22.0 for Windows (SPSS Inc. Chicago, IL, USA) was used for statistical analysis. All the results were reported as mean ± standard deviation (SD). The Kolmogorov–Smirnov test was used to confirm the normality of all the data. Independent t-test, χ^2^ test, or Mann–Whitney U test was used for baseline comparison of demographic characteristics, questionnaire data, and QEEG results between ASMR and BB groups. We examined within-group differences by using the paired t-test. Cohen’s *d* was calculated to estimate the effect size. Repeated measures analysis of variance (RM ANOVA) was used to confirm group-by-time interaction. For analysis of QEEG data, spectral analysis was done by fast Fourier transform. To normalize data distribution, absolute power was log-transformed. Relative power was chosen as a first principal analysis to reduce individual patient variation and bias between two recordings. We compared within-group differences of QEEG using the paired t-test in each group, and group-by-time interaction of all the QEEG power using RM ANOVA. All significance tests were two-sided and the *p*-value was set at < 0.05.

## Data Availability

The datasets of current study are available from the corresponding author on reasonable request.
